# Modulation of Pathways Underlying Distinct Cell Death Mechanisms in Two Human Lung Cancer Cell Lines in Response to S_N_1 Methylating Agents Treatment

**DOI:** 10.1371/journal.pone.0160248

**Published:** 2016-07-28

**Authors:** Olga Papadodima, Panagiotis Moulos, Aggeliki Koryllou, Georgia Piroti, Fragiskos Kolisis, Aristotelis Chatziioannou, Vasiliki Pletsa

**Affiliations:** 1 Institute of Biology, Medicinal Chemistry and Biotechnology, National Hellenic Research Foundation, 11635 Athens, Greece; 2 Institute of Molecular Biology and Genetics, Biomedical Sciences Research Centre ‘Alexander Fleming’, 16672 Vari, Greece; 3 Laboratory of Biotechnology, School of Chemical Engineering, National Technical University of Athens, 15780 Athens, Greece; 4 Enios Applications Private Company, 25 Al.Pantou str., 17671 Athens, Greece; University of Kansas Medical Center, UNITED STATES

## Introduction

Despite advances in the understanding of tumor biology in recent years, lung cancer remains the leading cause of cancer death worlwide. Non small cell lung cancer (NSCLC; adenocarcinoma, squamous carcinoma, and large cell carcinoma) is the most common type, representing more than 80% of lung cancer diagnoses. Clinical improvements following treatment with traditional chemotherapy have reached a plateau in patients with NSCLC and 5 year survival still remains at 15%. Moreover, the molecularly targeted therapies having already progressed to clinical trials have not proved successful [1,2].

Although NSCLC is very often extremely resistant to chemotherapeutic agents, chemotherapy remains an established treatment for advanced NSCLC. Platinum-based chemotherapy frequently combined with other agents and/or approaches remain the standard in the first-line treatment of patients with advanced NSCLC [3,4]. Methylating agents such as temozolomide, an oral S_N_1 methylating agent which crosses the blood brain barrier achieving effective concentrations in the Central Nervous System (CNS), have already been used combined with platinum-based regimen and whole brain radiotherapy as prophylaxis against brain metastasis in NSCLC and/or as second-line treatment in NSCLC patients with brain metastases [5–8].

Methylating agents of S_N_1 type, including temozolomide (TMZ), procarbazine, dacarbazine and streptozotocine, constitute a widely used class of anticancer drugs. These DNA damaging agents are highly cytotoxic, mutagenic, recombinogenic and clastogenic inducing about a dozen DNA methylation adducts [9]. Among them, O^6^-methylguanine (O^6^-meG), induced in small amounts (maximally 8% of total methylation products), is the most biologically significant lesion; it mispairs with thymine during DNA replication finally generating G to A transitions in the second round of replication. The DNA mismatches are recognized by the Methyl-directed Mismatch Repair (MMR) system that removes the mis-incorporated base opposite the O^6^-meG lesion. Repair resynthesis of DNA leads to reinsertion of thymine opposite O^6^-meG and re-initiation of futile mismatch repair. The DNA double-strand breaks (DSBs) resulting from this process are thought to initiate a series of events including prolonged G2 arrest leading to cell death [9]. A critical factor influencing the cellular response to methylating agents is O^6^-methylguanine- DNA methyltransferase (MGMT), the DNA repair protein that stoichiometrically and selectively removes methyl lesions from the O^6^ position of guanine and returns the DNA to its pre-lesioned state [10]. Pre-replicative repair by MGMT as well as post-replicative MMR determine the level of methylating agent-induced genotoxicity and cell death [11–13].

Chemotherapeutic agents inducing DNA damage, such as S_N_1 methylating agents and cisplatin, may activate cell death by apoptosis or necrosis. They could also induce autophagy, senescence or mitotic catastrophe, which may then be followed by apoptosis or necrosis [13–15]. The molecular basis underlying the decision- making process is currently the subject of intense investigation because a deeper understanding of how a given chemotherapy affects all of the signalling pathways involved in cell death is highly relevant in order to develop more effective therapeutics.

Despite their use in combination therapies, the effect of S_N_1 methylating agents on human NSCLC has not been studied thoroughly. We thus investigated the mechanism of the cell death induced by a model S_N_1 methylating agent, N-methyl-N-nitrosourea (MNU) in two human NSCLC cell lines, A549 (p53^wt^) and H157 (p53^null^) [13] through a time course gene expression profiling study 24, 48 and 72 hours after treatment. The list of differentiated genes, biological processes and cellular pathways were identified using appropriate bioinformatics tools and the results were further validated through RT-PCR of selected genes. MNU induced cell death through distinct responses at the gene expression level in the above cell lines. Our results overall support the use of S_N_1 methylating agents in platinum-based combination regimen against advanced NSCLC.

## Materials and Methods

### Cell culture and treatment

The human NSCLC lines NCI-H157 [p53^null^] and NCI-A549 [p53^wt^] were grown and treated as previously described [13]. Cells were harvested at 24, 48 and 72 h post MNU treatment and cytotoxicity and clonogenic cell survival were assayed as previously described [13]. DMSO treated cells (0.1% v/v) were used in all cases as controls. All assays were carried out in triplicate.

### RNA Extraction Step

RNA extraction was performed using the Trizol Plus Purification Kit (Invitrogen) according to the manufacturer’s instructions. On-column DNA digestion (RNase-Free DNase Set, Qiagen) was used to ensure the absence of DNA from the samples. The quantification and quality analysis of RNA was performed on a Bioanalyzer 2100 (Agilent, Santa Clara, California).

### Microarray Hybridization and Data Analysis

Synthesis of cDNA and biotinylated cRNA was performed with the Illumina TotalPrep RNA Amplification Kit (Illumina, San Diego, California) using 500 ng of total RNA. Hybridization was performed onto Illumina HumanWG-6_V3 Expression BeadChips according to manufacturer’s instructions. Three biological replicates for each condition were used, except for two cases, namely A549 cells treated with MNU for 72h and H157 cells treated with DMSO for 72h, since the isolated RNA did not meet the quality control criteria and the analysis was carried out with two replicates. The raw data were analyzed using GeneArmada software [16]. Briefly, background corrected values were log2 transformed and normalized using Quantile method. Probesets with a detection *p*-value > 0.01 in all tested conditions were not considered for further analysis. Significantly differentiated probe sets in MNU-treated as compared to DMSO-treated cells were identified by *t*-test by applying the following thresholds: *p-*value < 0.05, False Discovery Rate (FDR) < 0.1 and a fold change >|1| in log_2_ scale. Regarding the analysis of the GSE6410 dataset downloaded from NCBI's Gene Expression Omnibus (GEO), GCRMA normalization was applied and statistical selection was based on t-test with thresholds: *p-*value < 0.05 and fold change >|0.5| in log_2_ scale. Statistical enrichment analysis based on Gene Ontology (GO) terms or KEGG pathways was performed using StRAnGER [17].

### Real-Time PCR Analysis (RT-qPCR)

500 ng of RNA of selected genes were reverse-transcribed using Superscript III (Invitrogen, Carsland, CA, USA) reverse transcriptase and Random hexamer primers according to the manufacturer’s instructions. Real-time PCR reaction was performed with SYBR Green I (iQ SYBR Green Supermix, Biorad, Hercules, CA, USA) on an iQ5 Real-Time PCR Detection System (Biorad, Hercules, CA, USA) using GAPDH as an internal control for normalization. All assays were performed in triplicate in a 25 μL reaction. Specificity of the amplified PCR product was assessed through a melting curve analysis. Data analysis was performed according to the 2^-ΔΔCt^ method. Primers were designed with Primer-BLAST [18] and one primer per pair was designed to span an exon-exon junction. The list of primers is provided in S1 Table.

### Caspase-1 Inhibition

The A549 (p53^wt^) cells were treated with a specific caspase-1 inhibitor (50 μM Caspase-1 inhibitor Ac-YVAD-cmk, InvivoGen, San Diego, California, USA) for 1 h together with MNU. The inhibitor was kept in the medium until cells were harvested and counted at 24, 48 72 and 96 h post MNU treatment. Cytotoxicity was assessed by Trypan Blue (Sigma–Aldrich; T8154) according to the manufacturer’s standard protocol as previously described [13].

### Western blot analysis

Total protein extracts were prepared using RIPA lysis buffer purchased from Santa Cruz. Cell lysates (40 μg protein) were resolved by SDS-PAGE, electrotransferred onto nitrocellulose and blocked by incubation in 5% nonfat dry milk in phosphate buffered saline for 1 h at room temperature. The nitrocellulose was incubated with primary antibody overnight at 4°C, followed by incubation with secondary antibody conjugated to horseradish peroxidase for 1 h at room temperature. Detection was achieved using ECL kit (Amersham Life Science, UK). Primary antibodies against the following proteins were used at the indicated dilutions: actin (MAB1501; Chemicon), 1:5000; p21 Waf1/Cip1(2947; cell signalling), 1:500; Caspase-1(p20) (AG-20B-0048; Adipogen Life Sciences), 1:500.

## Results and Discussion

Although platinum-based chemotherapy remains an established treatment for advanced NSCLC, it is very often combined with other agents and/or approaches to further enhance the effectiveness of the therapy [3,4]. Due to their genotoxic properties S_N_1 methylating agents such as temozolomide have already been used in combination therapies [5–8], however, their effect on human NSCLC has not been studied thoroughly.

In an effort to elucidate the molecular mechanism of cell death induced by S_N_1 methylating agents, we had previously investigated the cytotoxic effect of equimolar cytotoxic doses of MNU, in the human NSCLC cell lines NCI-H157 [p53^null^] and NCI-A549 [p53^wt^]. In accordance with all evidence concerning Temozolomide—a methylating agent having demonstrated anti-tumour activity against a broad range of tumour types [5–8, 19–22]—our data clearly demonstrated that S_N_1 methylating agents, like MNU, effectively kill NSCLC cell lines, implying their possible use as chemotherapeutic agents against this type of cancer, however, the mode of the cell death induced is cell type dependent [13]. Both cells lines possess a functional MMR system and moderate levels of MGMT (A549:150 fmol MGMT/mg protein, H157:160 fmol MGMT/mg protein). The dose effectively killing 90–100% of cells 10 days after treatment (Lethal Dose_90_−_100_ = 200 μg/ml) induces a variety of cytotoxic lesions, other than O^6^-methylguanine [23]. Still, the role of the DNA repair protein MGMT is critical since its depletion by O^6^-benzylguanine treatment lowers the effective cytotoxic dose by an order of magnitude (LD_90-100_ = 20 μg/ml) in both cell lines [13].

Guided by our previous results, we undertook the current gene expression profiling study to further investigate the mechanism of MNU-induced cell death.

### Statistically significant differentiated genes

To obtain the transcriptomic profile of A549 and H157 cells in response to MNU, cells were treated with isotoxic doses of MNU and total RNA was isolated at 24, 48 and 72h after treatment. The WG-6 Illumina platform was used for microarray analysis and the complete dataset has been deposited in GEO with accession number GSE65933. Statistical analysis at each time point, coupled with fold change filtering, resulted in 899 significantly differentiated genes in A549 and 541 genes in H157 cells (S2 and S3 Tables).

**Fig 1:**
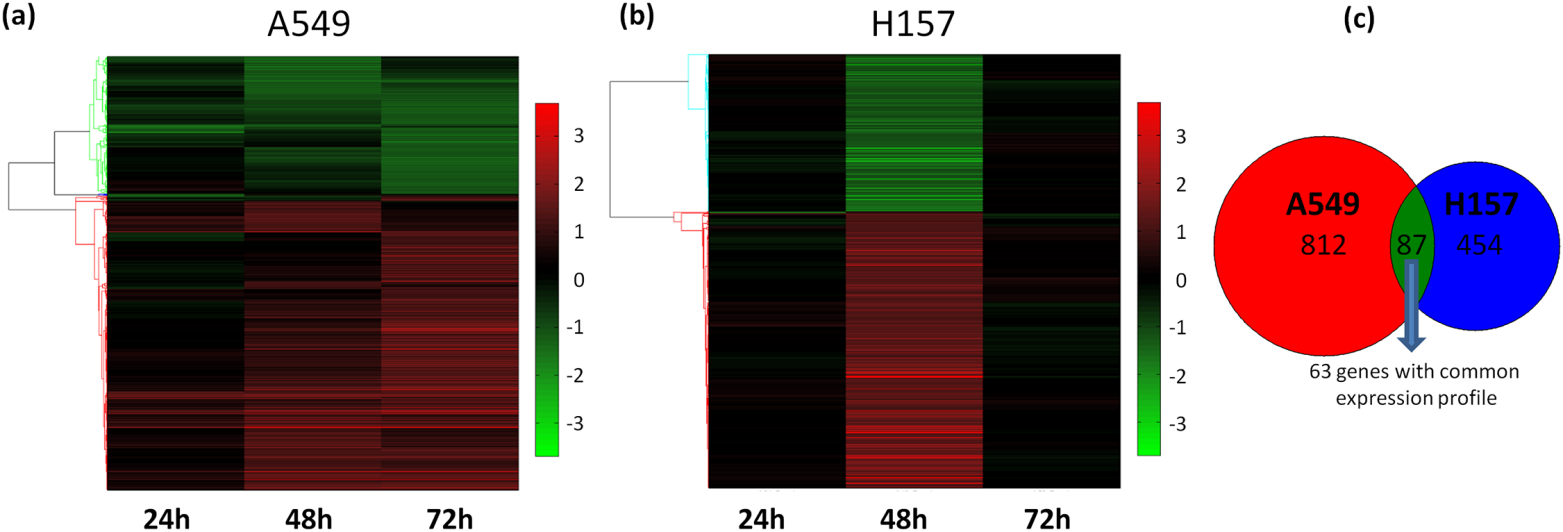
Statistically significant differentiated genes. Hierarchical clustering of the expression fold change values, as compared between MNU and DMSO treated cells after 24, 48 and 72h of treatment. The genes found as significantly differentiated in A549 **(a)** and H157 cells **(b)** are shown, where red color indicates up-regulated genes and green indicates down-regulated genes. **(c)** Venn diagram showing the total number of genes found as significantly differentiated in A549 and H157 cells, after MNU treatment. Among the 87 common genes, 63 share a common expression profile (up- or down-regulation) in both cell lines.

To analyze the MNU induced alterations at the transcriptome of the two NSCLC cell types, the lists of differentiated genes of A549 and H157 cells were subjected to hierarchical clustering analysis depicted in Fig 1a and 1b, respectively. Major changes began to appear after 48 hours since efficient killing by S_N_1-methylating agents requires DNA replication and cell division [9,11,13]. The expression profile in A549 cells seemed constant between 48 and 72h of treatment. In contrast, in H157 cells the observed gene expression alterations were transiently peaking at 48h, returning to the control levels at 72h.

Among the differentiated genes, we found 87 genes in common between A549 and H157 cells (Fig 1c) of which 63 showed the same direction in gene expression alteration; 36 were up-regulated in both cell lines and 27 were down-regulated after MNU treatment, as compared to control cells. Using the 27 genes down-regulated in both cell lines as input for functional analysis based on Gene Ontology (GO) terms, the biological processes significantly modified were mostly related to cell cycle control, namely mitotic cell cycle, nuclear division and chromosome organization. This is in agreement with the fact that both cell lines incurred cell death through prolonged G2 arrest following MNU treatment [13].

#### Cell-specific statistically significant differentiated genes in A549 cells

Of the 899 genes affected in A549 cells, 812 were cell-type specific. The top differentiated genes in terms of fold change in their expression, as compared between MNU treated and non-treated cells, are listed in Table 1.

**Table 1:** Top up- and down-regulated genes in A549 cells after MNU treatment.

		Fold Change*		
Symbol	Description	24h	48h	72h
CEACAM1	carcinoembryonic antigen-related cell adhesion molecule 1	1.77	3.71	3.66
GPR172B	G protein-coupled receptor 172B	2.22	3.37	3.56
TP53INP1	tumor protein p53 inducible nuclear protein 1	1.87	3.31	2.95
TRIM22	tripartite motif-containing 22	1.62	3.17	3.49
ABCA12	ATP-binding cassette, sub-family A, member 12	1.41	2.88	2.54
ACTA2	actin, alpha 2, smooth muscle, aorta	2.09	2.75	2.56
SPATA18	spermatogenesis associated 18 homolog (rat)	2.09	2.70	2.98
CDKN1A	cyclin-dependent kinase inhibitor 1A (p21, Cip1)	1.76	2.66	2.97
ALOX5	arachidonate 5-lipoxygenase	1.02	2.56	3.36
CXCR4	chemokine (C-X-C motif) receptor 4	0.57	2.50	2.07
SUGCT	chromosome 7 open reading frame 10	0.99	2.50	3.01
FAS	Fas (TNF receptor superfamily, member 6)	1.81	2.49	1.83
SULF2	sulfatase 2	1.77	2.46	1.73
CYGB	Cytoglobin	1.87	2.45	2.93
GDF15	growth differentiation factor 15	2.42	2.43	1.95
RN7SK	RNA, 7SK small nuclear, non-coding RNA	0.06	-2.95	0.39
RNU6ATAC	RNA, U6atac small nuclear (U12-dependent splicing)	-0.35	-2.37	-0.46
CALB2	calbindin 2, transcript variant CALB2c	-1.09	-1.94	-1.64
DCLK1	Doublecortin-like kinase 1	-1.49	-1.92	-0.35
FOSL1	FOS-like antigen 1	-0.83	-1.90	-0.18
NPTX1	neuronal pentraxin I	-1.10	-1.78	-0.63
SNORA62	small nucleolar RNA, H/ACA box 62, small nucleolar RNA	-0.13	-1.64	-0.70
IRF2BP1	interferon regulatory factor 2 binding protein 1	0.15	-1.58	-0.21
GAL	galanin prepropeptide	-0.37	-1.53	-1.29
RPPH1	ribonuclease P RNA component H1	-0.68	-1.52	-0.61
ANPEP	alanyl (membrane) aminopeptidase	-1.10	-1.48	-0.15
LRRC26	leucine rich repeat containing 26	-0.33	-1.46	-1.05
RPRML	reprimo-like	-0.49	-1.43	-0.69
H1F0	H1 histone family, member 0	-0.83	-1.42	-0.42
RAC3	ras-related C3 botulinum toxin substrate 3	-0.42	-1.41	-0.75

* Expression fold changes, as compared between MNU treated and untreated cells, are shown for 24, 48 and 72h after treatment and genes are sorted according to the 48h values.

**Fig 2:**
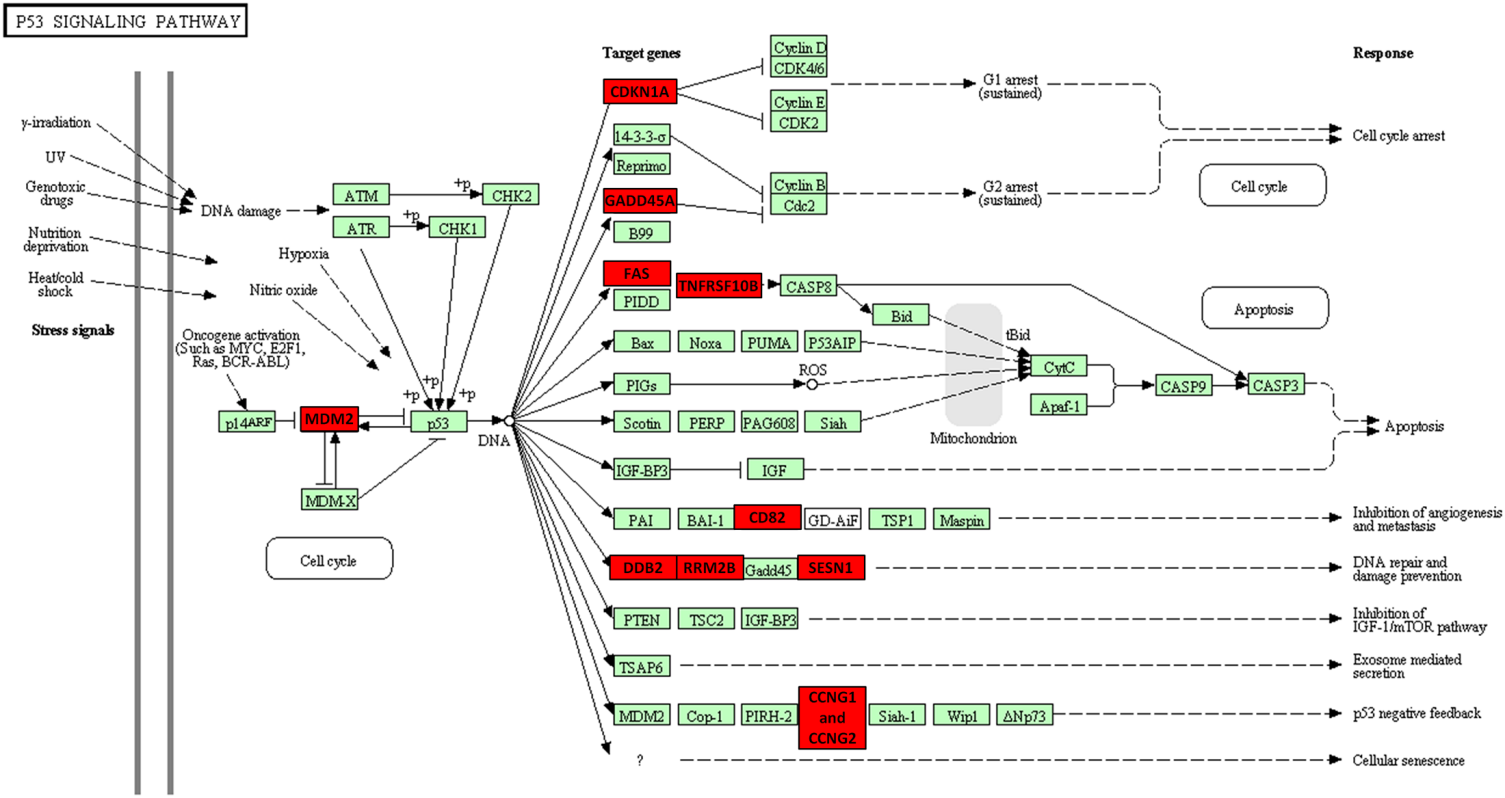
StRAnGER pathway analysis. StRAnGER pathway analysis exploiting KEGG database, based on the up-regulated genes after MNU-treatment in A549 cells. The P53 signaling pathway is ranking at the top of the significantly over-represented pathways. Genes found as significantly up-regulated are shown in red.

The MNU-induced cell death in A549 cells (p53^wt^) exhibited a molecular profile characterised by up-regulation of a significant number of p53 downstream target genes; as validated through pathway analysis shown in Fig 2, several targets of the p53 pathway implicated in cell cycle regulation and arrest (*p21*, *GADD45*), apoptosis (*FAS*, *TNFRSF10B*, *TP53I3*, *ZMAT3*), inhibition of angiogenesis and metastasis (*SERPINE1*, *CD82*), DNA repair and damage prevention (*DD82*, *RRM2B*, *SESN1* and *SESN2*) and p53 negative feedback (*MDM2*, *CCNG1*, *CCNG2* and *PPM1D*) were detected. (Table 1 and S1 Table). This is in agreement with previous biochemical data where cell death was accompanied by p53 phosphorylation/stabilization, p21 induction and degradation of Cdc25A at the post–translational level [13]. It is worth mentioning that there seems to be a cell-specific profile regarding DNA damage response since the vast majority of the above mentioned p53 downstream target genes were also up-regulated following treatment of A549 cells with cisplatin [24].

Furthermore, three genes related to cell death mechanisms with anti-tumor properties, namely Carcinoembryonic antigen-related cell adhesion molecule 1 (*CEACAM1*), E-cadherin (*CDH1)* and Caspase-1 (*CASP1*), were also significantly up-regulated (Table 1). Regarding CEACAM1 in NSCLC, a recent study comparing NSCLC patients versus healthy donors concluded that it might be a useful marker in early diagnosis [25], still the emerging picture of its involvement in cancer remains very complex [26–28]. Our finding is in agreement with previous studies considering *CEACAM1* as a tumor suppressor gene, supporting the association of its expression with apoptosis in early colon tumour lesions in vivo [26,27]. E-cadherin *(CDH1)* is a tumour suppressor gene coding for a protein with a strong anti-metastatic and anti-invasive role [29]. Caspase-1 (*CASP1*) has been reported to mediate sensitisation to cisplatin and gamma-radiation both in vitro and in vivo [30]. Moreover, its up-regulation has been suggested to induce pyroptosis, a caspase-1-dependent type of programmed cell death, not depending on apoptotic caspases [31,32]. This is again in agreement with our previous biochemical data in A549 cells (p53^wt^), where a caspase-independent mode of cell death was observed [13].

Among the biological processes mostly affected within the group of the up-regulated genes were cell adhesion (26 genes), negative regulation of cell proliferation (16 genes) and immune response (20 genes). The complete list is given in S4 Table.

Interestingly, three non-coding RNAs are in the top 15 list of the down-regulated genes. The first, being 7SK small nuclear, non-coding RNA (*RN7SK*), negatively regulates the positive transcription elongation factor P-TEFb while it also controls the high-mobility group protein HMGA1-induced transcription initiation and chromatin remodeling. There is evidence that 7SK RNA complexes contain simultaneously HMGA1 and P-TEFb, thus establishing gene-dependent plasticity between HMGA1 chromatin remodeling and transcription initiation and P-TEFb transcription elongation [33]. *HMGA1* was also down-regulated (S2 Table). The degree of *HMGA1* up-regulation correlates with tumor malignancy and metastatic potential [34]. The involvement of these non-coding RNAs definitely deserves further investigation. GO analysis, based on the down-regulated genes (S5 Table), indicated that the biological processes mostly affected were DNA repair (20 genes), DNA replication (21 genes) and mitosis (25 genes).

In a study having investigated the effect of cisplatin in a panel of NSCLC cell lines in relation to a series of different end-points, among them sensitivity/resistance, apoptosis and gene expression changes, A549 was found to be the most resistant cell line not undergoing apoptosis. The cisplatin-induced gene expression changes were analyzed by microarray analysis and raw data are deposited in GEO [24]. We downloaded the relevant dataset, in order to compare the cisplatin and MNU induced gene expression alterations. In the case of cisplatin treatment, 97 genes were found as significantly differentiated, while 32 of them, depicted in S6 Table, showed the same expression profile as the one observed in our study following MNU treatment. Functional analysis on these 32 genes revealed as significantly enriched the terms “p53 pathway” (10 genes) and “cell cycle” (13 genes). It is important to note that the number of genes altered after MNU treatment (899 genes, S2 Table) is much higher than that observed with cisplatin. Although the differences in the experimental conditions, including time of treatment and the microarray platform used for the analysis, could affect the total number of differentiated genes, it is noteworthy that an important number of genes with altered expression are found only after MNU treatment. Among them, *CASP1* and DNA repair genes, mentioned above, are of particular interest since they indicate candidate targets affected only by MNU.

Caspase-1 activation is related to pyroptosis, a form of cell death that does not have the features of apoptosis. Pyroptosis is morphologically characterized by the formation of plasma membrane pores as well as final rupture of the membrane, resulting from caspase-1-mediated processes. The process is initiated by ligation of TLRs (toll-like receptors) on the plasma membrane and NLRs (NOD-like receptors) in the cytosol to PAMPs (pathogen associated molecular patterns)/DAMPs (damage associated molecular pattern molecules), followed by the formation of intacellular large signalling complexes called inflammasomes. Four types of inflammasomes containing NLRP1, NLRP3, IPAF/NLRC4 and AIM2 respectively, along with caspase-1 as the common effector molecule have been reported. All inflammasomes contain a CARD (caspase activation and recruitment domain) or pyrin domain (PYD). The resultant conversion of caspase-1 into its active form ignites a panel of cellular responses including the secretion of pro-inflammatory cytokines, such as IL-1β (interleukin-1β), and ultimate cell lysis [35].

Upon MNU-induced cell death (Table 1 and S2 Table), genes as *TRIM22* (tripartite motif-containing 22), *PYCARD* (PYD and CARD domain containing, transcript variant 1), *TLR3*, *TLR4* and *IL-1β* are up-regulated. The tripartite motif-containing families (TRIM) are Pattern Recognition Receptors (PRRs) known to mediate activation of caspase-1 by DAMPs, while PYCARD is part of the AIM2 inflammasome which is able to recognize cytoplasmic double-stranded DNA (dsDNA) that is not necessarily of microbial origin. Although distinct from NLRs, AIM2 has a pyrin domain that allows it to interact with the adaptor protein ASC (apoptosis-associated speck-like protein containing a CARD) to form the inflammasome [36, 37]. There is evidence demonstrating the links between DNA damage-induced cell death and inflammation, regarding alkylating DNA damage in particular [38]. When necrosis occurs, as a consequence of severe DNA damage, plasma membrane integrity is lost, thus, allowing escape of intracellular material from the cell. Such a prototypical DAMP derived from necrotic cells is the chromatin–associated protein high mobility group box 1 (HMGB1) however, DNA could function as a DAMP as well when released outside the nucleus. The mechanism by which DNA damage causes inflammation is complex, often involving kinases activated by DNA single- or double-strand breaks, or DNA fragments recognized by pattern-recognition receptors inducing downstream inflammatory signaling [38, 39]. Furthermore, new insights into the role of the DNA Damage Response (DDR) in innate immunity provide new clues as to the essential character of p53 [40]. The results of the gene expression profile analysis in A549 cells suggest that a considerable part of the MNU-induced cell death may be due to pyroptosis implying a cross-talk between DNA Damage Response (DDR) and innate immunity which requires a thorough investigation in the case of S_N_1 methylating agent-induced DNA damage.

As regards the DNA repair genes affected, the *Ribonucleotide reductase M2 B* (*RRM2B*), coding for an enzyme playing a vital role in DNA synthesis and repair, is up-regulated while *X-ray repair cross complementing group 3 (XRCC3)* and *Breast Cancer susceptibility gene 1* (*BRCA1)* both coding for proteins involved in the double-strand break repair (DSBR) pathway are down-regulated.

The DNA repair genes are suspected to be related to the survival of lung cancer patients due to their possible influence on DNA repair capacity. Still, evidence supporting their genetic involvement in lung cancer risk and survival remains inconclusive [41]. RRM2B is involved in p53-dependent cell cycle regulation under DNA-damage conditions and can be regulated by p53, p73 and FOXO3. Overexpression of *RRM2B* and/or *FOXO3*, which is also up-regulated in our system (S2 Table), can suppress metastasis and inhibit proliferation in certain cancer cells [42]. The protein encoded by *XRCC3* is a member of the double-strand break (DSB) repair pathway and plays a direct role in homologous recombination that is important for the integrity of chromosome and repair of damaged DNA. A polymorphism in codon 241 (Thr to Met, rs861539 C>T) of *XRCC3* has been linked to an increased level of DNA adducts in healthy people and associated with risk of lung cancer [43]. Results from a recent meta-analysis showed that this *XRCC3* polymorphism can predict good response to platinum-based chemotherapy in patients with advanced NSCLC, especially in Caucasian population, while there is no significant association with survival of NSCLC [44,45]. On the other hand, apart from its role in transcriptional regulation, cell cycle control, ubiquitination, apoptosis, and mitotic spindle assembly, BRCA1 has a prominent role in DNA repair and regulation of genome stability. BRCA1 is involved in platinum adducts removal as a component of transcription-coupled Nucleotide Excision Repair (NER) and homologous recombination repair pathways during the repair of double-strand breaks. Experimental and clinical studies have demonstrated that *BRCA1* mRNA expression level is differentially associated with the response to chemotherapeutic drugs and ionizing irradiation and low *BRCA1* mRNA is correlated with cisplatin sensitivity in patients with non-small cell lung cancer [46,47].

Our results in A549, regarding the above mentioned DNA repair genes as well as the DNA repair capacity which is down-regulated as a whole following MNU treatment, support the use of S_N_1 methylating agents combined with platimum-based regimen against NSCLC; in a p53 context, they clearly induce a caspase1-dependent mode of cell death affecting the expression of critical DNA repair genes, different from the ones affected by cisplatin treatment. Therefore, the use of S_N_1 methylating agents could significantly enhance chemosensitivity and reverse chemoresistance in platinum-based first and/or second-line treatment.

#### Cell-specific statistically significant differentiated genes in H157 cells

In this case, 454 of the 541 genes affected were cell-type specific. The top differentiated genes in terms of fold change in their expression, as compared between MNU treated and non-treated cells, are listed in Table 2.

**Table 2:** Top up- and down-regulated genes in H157 cells after MNU treatment.

		Fold Change*		
Symbol	Description	24h	48h	72h
TSC22D3	TSC22 domain family, member 3	-0.14	3.95	-0.34
SCG5	secretogranin V	0.45	3.91	-0.16
MAOA	monoamine oxidase A, nuclear gene encoding mitochondrial protein	-0.12	3.84	-0.35
RRAGD	Ras-related GTP binding D	0.01	3.78	0.01
VCX	variable charge, X-linked	0.06	3.67	-0.18
VCX-C	variably charged X-C	0.29	3.22	-0.29
GAGE4	G antigen 4	0.16	3.15	-0.35
GAGE5	G antigen 5	0.37	3.14	0.12
GAGE12G	G antigen 12G	0.34	3.13	-0.01
STC2	stanniocalcin 2	0.01	3.12	-0.15
GAGE12I	G antigen 12I	0.21	3.10	-0.11
NPTX1	neuronal pentraxin I	0.43	2.86	-0.33
GAGE6	G antigen 6	-0.19	2.79	-0.14
MMP3	matrix metallopeptidase 3	0.10	2.71	0.06
SPANXN1	SPANX family, member N1	-0.15	2.70	-0.21
IFI27	interferon, alpha-inducible protein 27	0.50	-4.52	-0.18
ALDH1A1	aldehyde dehydrogenase 1 family, member A1	-0.18	-3.55	-0.12
IFIT1	interferon-induced protein with tetratricopeptide repeats 1	-0.63	-3.12	0.06
OLR1	oxidized low density lipoprotein (lectin-like) receptor 1	0.27	-3.03	0.29
CCL20	chemokine (C-C motif) ligand 20	-0.38	-2.98	0.56
SCD	stearoyl-CoA desaturase (delta-9-desaturase)	-0.20	-2.95	0.52
CCL5	chemokine (C-C motif) ligand 5	-0.64	-2.88	0.00
CXCL2	chemokine (C-X-C motif) ligand 2	-0.46	-2.85	0.21
PTGES	prostaglandin E synthase	0.23	-2.72	0.37
CSF3	colony stimulating factor 3	-0.33	-2.65	-0.04
TGM2	transglutaminase 2 (C polypeptide, protein-glutamine-gamma-glutamyltransferase)	-0.06	-2.13	0.27
ANP32AP1	acidic nuclear phosphoprotein 32 family, member A, non-coding RNA	0.54	-2.07	0.11
CCL2	chemokine (C-C motif) ligand 2	-0.06	-2.06	0.08
DHCR7	7-dehydrocholesterol reductase	-0.46	-2.05	0.19
ACAT2	acetyl-Coenzyme A acetyltransferase 2	-0.15	-1.99	0.07

* Expression fold changes, as compared between MNU and DMSO treated cells, are shown in log_2_ scale for 24, 48 and 72h after treatment. Genes are sorted according to the 48h values.

The molecular profile of the cell death induced by MNU in H157 cells (p53^null^) differed considerably from the one observed in A549 (p53^wt^), in line with previous biochemical analysis [13]. Among the top 15 up-regulated genes, *TSC22D3* (*GILZ*: glucocorticoid-induced leucine zipper), a transcriptional regulator stimulated by glucocorticoids and interleukin 10 was the first. GILZ, a protein ubiquitously expressed, regulated mainly by steroid hormones, appears to play a key role in the anti-inflammatory and immunosuppressive effects of IL-10 and regulates cell cycle, apoptosis, and differentiation [48].

*GAGE* and *VCX/Y* gene family members were also detected. This is a very interesting finding as many aberrantly expressed antigens are currently being targeted as therapeutic cancer vaccines. Tumor-specific antigens recognized by autologous T lymphocytes are encoded by genes, including those of the *MAGE* (up-regulated also as shown in S3 Table), *BAGE*, and *GAGE* gene families, expressed in a significant fraction of various tumors, but not in normal adult tissues, except for testis where they appear to be expressed in germ cells [49,50]. Cancer/testis (CT) antigens are considered promising targets for cancer immunotherapy. *VCX/Y* genes, mostly located on the X chromosome, were recently identified as novel CT antigens. The expression levels of VCX/Y mRNAs were reduced in normal, immortalized lung cell lines compared to lung cancer ones. Reactivity against the protein product of the *VCX3A* gene was observed in approximately 20% of lung adenocarcinoma and 35% of squamous cell carcinoma (SCC) tumors using tissue microarrays, whereas no expression was detected in normal lung tissues [51]. The fact that MNU-induced cell death was accompanied by significant CT up-regulation could be exploited for the development of combinatory protocols including chemotherapy as well as immunotherapy [52] against certain forms of lung cancer. During the last decade, there has been an intensive effort to understand the way cancer interacts with the immune cells and overcome the limitations of conventional therapeutic strategies. Furthermore, GO analysis of the up-regulated genes (S7 Table), indicated that among the biological processes mostly affected were regulation of transcription (36 genes), apoptosis (13 genes) and inflammatory response (11 genes).

Regarding the down-regulated genes, the Interferon alpha-inducible protein 27 (*IFI27*), found to be up-regulated in various cancers [53] and Chemokines (*CC*, *CXC* subfamilies) were detected high in the ranking list. Recently, the importance of chemokines and chemokine receptors in inflammation associated with carcinogenesis has been highlighted. Increasing evidence suggests that chemokines are produced by tumor cells and also by cells of the tumor microenvironment and besides having effects on tumor cell proliferation, angiogenesis and metastasis, chemokines also appear to modulate senescence and cell survival [54]. The down-regulation of all the above genes, in the process of the cell death induced, further enhanced the methylating agent (MNU) effect in this particular cellular background, and is in line with the apoptotic mode observed in H157 cells in the course of the biochemical analysis [13]. Among the biological processes mostly affected by the down-regulated genes were cholesterol biosynthetic process (6 genes) and cell cycle (13 genes) (S8 Table).

### RT-PCR validation and correlation with microarray data

**Fig 3:**
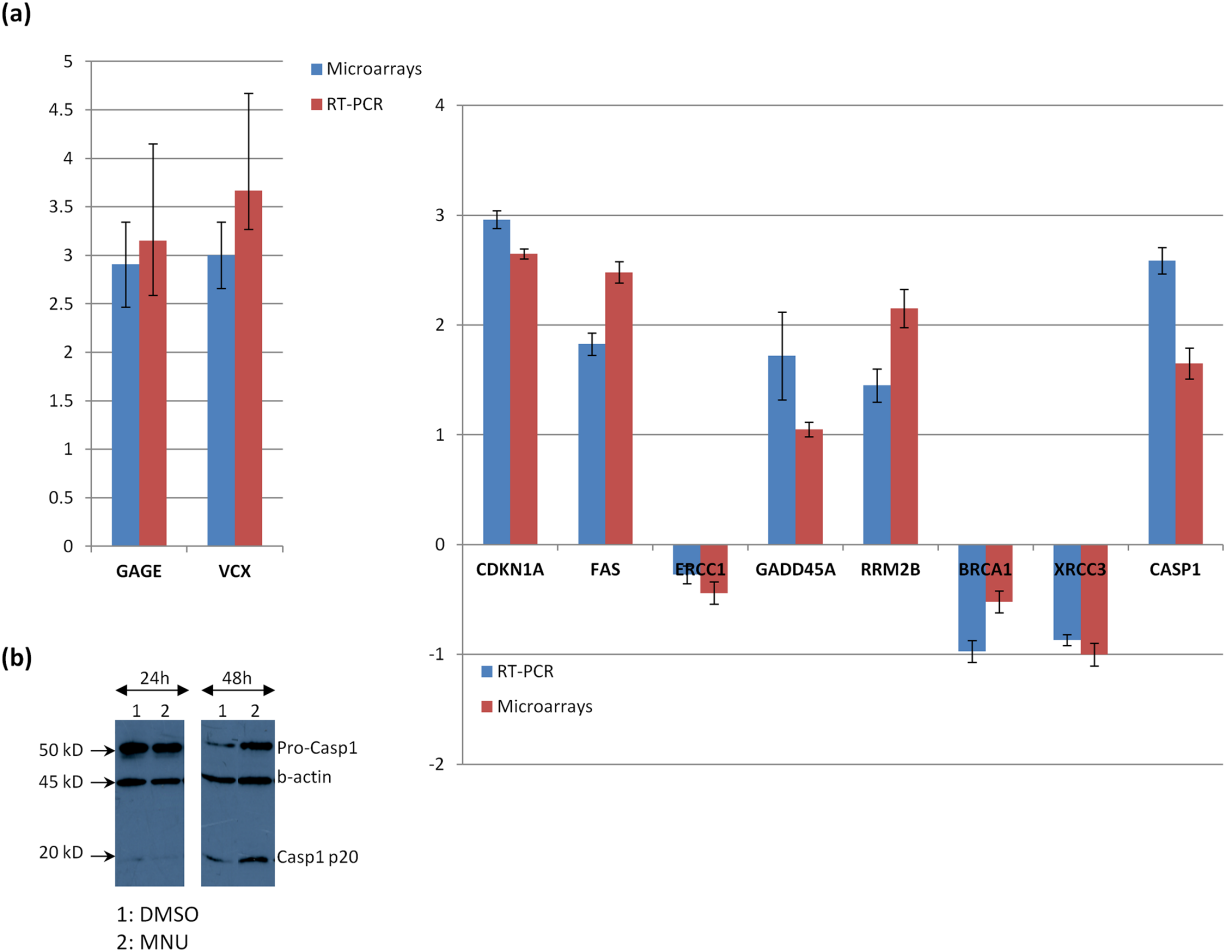
Validation of Microarray data. **(a)** Validation of microarray data by RT-PCR. Fold change in expression (in log_2_ scale) of the selected genes, as compared between MNU and DMSO treated cells, as mentioned by qRT-PCR and microarrays. Values represent the mean of three independent reactions and error bars the standard deviations. *GAPDH* was used as an internal control for data normalization. All expressions were statistically significantly different between MNU and DMSO treated cells, except those of ERCC1 which was used as internal control. **(b)** Immunoblot of A549 cell extracts (40 μg) with a-caspase-1 antibody. Cells were either DMSO (0.1% v/v) or MNU (200 μg/ml) treated and maintained in culture for the indicated length of time. The relative abundance of total protein applied was measured by using as control the amount of actin as assessed by a-actin on the same blot.

The validity of the microarray data was tested by quantitative RT-PCR 48 h after MNU treatment, when gene expression alterations were mostly pronounced in both cell types. In the case of A549 cells, we selected *CDKN1A*, *FAS and GADD45A* related to the p53-dependent cell death pathway as well as *RRMB2B*, *XRCC3*, *BRAC1* and *CASP1* exclusively affected by MNU, related to DNA repair and cell death mechanisms. *ERCC1* (excision repair cross-complementation group 1), a DNA repair gene essential in the Nucleotide Excision Repair pathway, potentially implicated in NSCLC prognosis [47,55] was used as an internal control, since its expression was approximately at the control levels at 48h. In H157 cells, we sought to test the expression of the *VCX/Y* and *GAGE* families since they dominated the top 15 up-regulated gene list. Based on the similarity among the members of the two families, primers were designed to hybridize to several different genes of each family and generate the same amplicon. MNU-induced changes in the expression of the selected genes as assessed by both microarray and RT-PCR methods are presented in Fig 3. In the case of microarrays, the presented values correspond to the expression fold change of *VCX* and *GAGE4* genes, which showed the highest expression alteration. Comparing the relative expression values obtained by microarrays and qRT-PCR, a good correlation was observed. Caspase-1 up-regulation upon MNU treatment was further confirmed at the protein level by western blotting (Fig 3).

### Caspase-1 Inhibition

**Fig 4:**
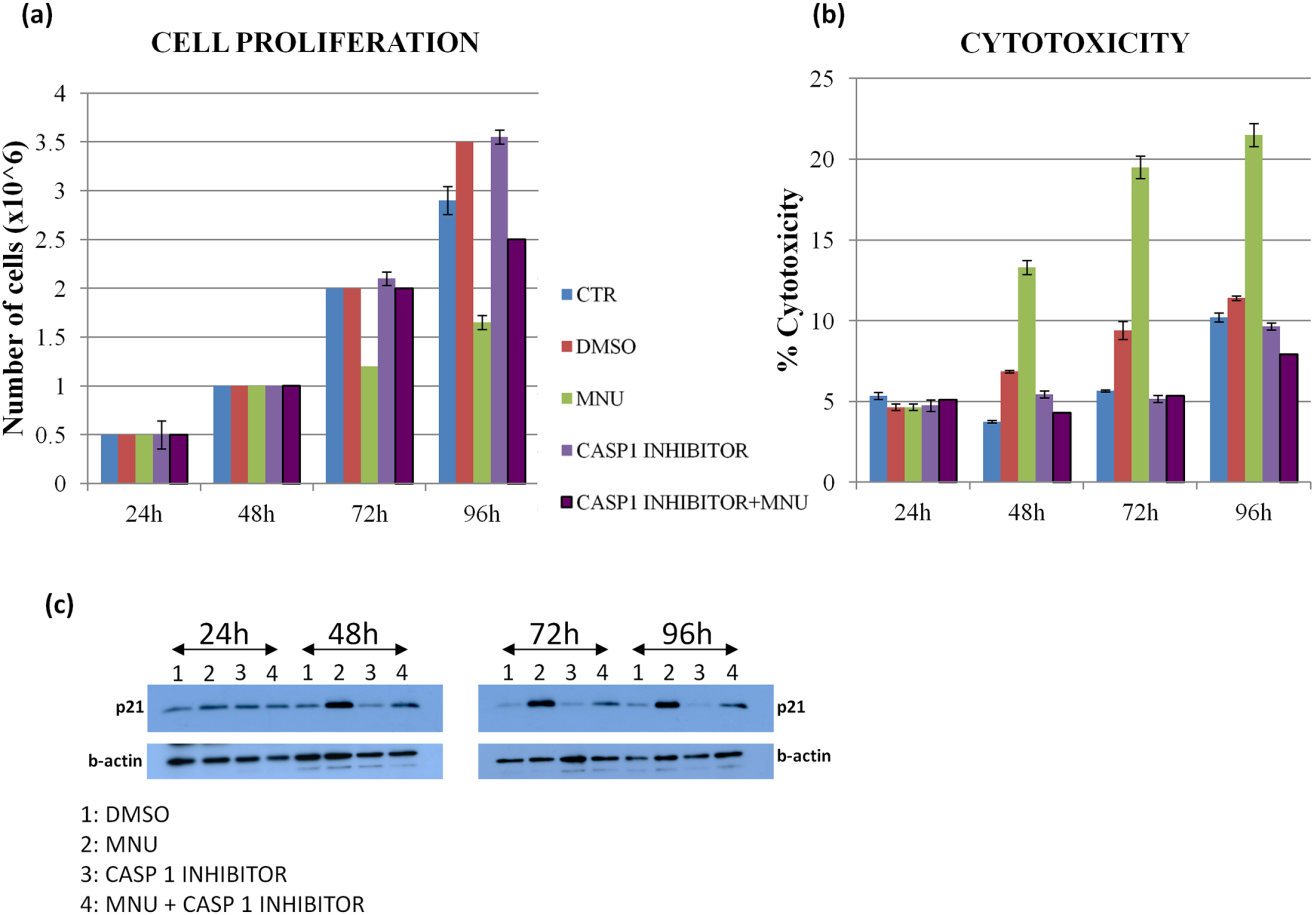
Effect of Caspase-1 Inhibition on MNU-induced cell death in A549 cells. **(a)** Cell proliferation in the absence or presence of caspase-1 inhibitor 24,48,72 and 96 hours post DMSO or MNU treatment. **(b)** Cytotoxicity of DMSO and MNU, in the absence or presence of caspase-1 inhibitor 24,48,72 and 96 hours post treatment. Values represent the mean of three independent experiments and error bars the standard deviations. **(c)** Immunoblot of A549 cell extracts (40 μg) with a-p21 antibody. Cells were either DMSO (0.1% v/v) or MNU (200 μg/ml) treated and maintained in culture for the indicated length of time. The relative abundance of total protein applied was measured by using as control the amount of actin as assessed by a-actin on the same blot.

In order to confirm that the MNU-induced cell death in A549 cells (p53^wt^) is, to a significant extent, due to pyroptosis, cells were treated with MNU in the absence or presence of the specific caspase-1 inhibitor (50 μM Caspase-1 inhibitor Ac-YVAD-cmk, InvivoGen, San Diego, California, USA). Cell viability and cytotoxicity were assessed 24, 48,72 and 96 h post MNU treatment. Efficient killing by S_N_1 methylating agents is a late event as it requires DNA replication and cell division [9,11,13], therefore, the cells were kept in culture the longest possible. Major changes appeared after 48 hours; MNU-induced DNA damage considerably inhibited cell proliferation and increased cytotoxicity at 72 and 96 hours (Fig 4a and 4b). The caspase-1 inhibition did not affect cell proliferation in the non-MNU treated cells, but it significantly delayed the MNU-induced cell death (Fig 4a and 4b). The impact of caspase-1 inhibition on the p53-dependent cell death pathway was further assessed by western blotting of p21 (Fig 4c), shown to be up-regulated in the gene expression profile analysis (Fig 2) and induced,at protein level, upon MNU treatment in our previous studies [13]. Caspase-1 inhibition significantly attenuated the induction of p21 upon MNU treatment indicating a cross-talk between the DDR and Pyroptosis pathway; our results overall suggest that upon MNU treatment a non-apoptotic programmed cell death is initially induced leading to cell lysis which consequently activates the inflammasome, caspase-1 dependent pyroptosis pathway.

## Conclusions

In A549 cells (p53^wt^), MNU treatment induced cell death through the up-regulation of p53 pathway genes accompanied by the down-regulation of genes affecting DNA repair, cell division, mitosis and transcription initiation, suggesting an alternative mechanism of programmed cell death, other than apoptosis, p53-and caspase-1- dependent. Caspase-1 inhibition significantly attenuated the MNU-induced, p53-dependent cell death, indicating the synergistic contribution of pyroptosis in the process. The results obtained support the use of S_N_1 methylating agents in combination with platimum-based regimen against NSCLC in view of a synergy that could significantly enhance chemosensitivity and/or reverse chemoresistance frequently observed in the case of cisplatin.

In H157 cells (p53^null^), the MNU-induced genes were mostly related to apoptosis and inflammatory response; furthermore, a pronounced up-regulation of several genes coding Cancer/testis (CT) antigens was observed, providing the possibility for the development of combinatory chemotherapy-immunotherapy protocols.

Our results, overall, indicate that the mode of cell death depends on the molecular profile of the cells which is highly relevant to tumour-type treatment favouring the currently accepted concept of targeted therapy through tailor-made protocols.

## Supporting Information

S1 Table List of primers used in RT-PCR. (XLSX)

S2 Table List of statistically significant differentiated genes in A549 cells. (XLSX)

S3 Table List of statistically significant differentiated genes in H157 cells. (XLSX)

S4 Table GO-analysis using as input the up-regulated genes after MNU-treatment of A549 cells. (XLSX)

S5 Table GO-analysis using as input the down-regulated genes after MNU-treatment of A549 cells. (XLSX)

S6 Table Common gene expression alterations induced by both cisplatin and MNU. (XLSX)

S7 Table GO-analysis using as input the up-regulated genes after MNU-treatment of H157 cells. (XLSX)

S8 Table GO-analysis using as input the down-regulated genes after MNU-treatment of H157 cells. (XLSX)
