# Correction: Modulation of Pathways Underlying Distinct Cell Death Mechanisms in Two Human Lung Cancer Cell Lines in Response to SN1 Methylating Agents Treatment

**DOI:** 10.1371/journal.pone.0194257

**Published:** 2018-03-08

**Authors:** The PLOS ONE Journal Staff

The Abstract section is missing. The publisher apologizes for the error. The Abstract can be viewed here:

## Abstract

Despite concerted research efforts, lung cancer remains the leading cause of cancer death worldwide, therefore, new therapeutic approaches are eagerly needed. Methylating agents of S_N_1 type constitute a widely used class of anticancer drugs, the effect of which on human non small-cell lung cancer (NSCLC) has not been adequately studied although frequently combined with platinum-based chemotherapy. We thus studied the effect of N-methyl-N-nitrosourea (MNU), a model S_N_1 methylating agent, on two human NSCLC cell lines; A549 (p53^wt^) and H157 (p53^null^). The mechanism of MNU-induced cell death was investigated through a time course gene expression profiling study, 24, 48 and 72h following treatment. Differentiated genes, biological processes and cellular pathways were identified using appropriate bioinformatics tools. The results were validated through RT-PCR of selected differentiated genes. The number of statistically significant differentiated genes, presenting a minimum of 2 fold alteration in their expression, was 920 for A549 and 541 for H157 cells. In both cell lines, the MNU-induced alterations in gene expression became prominent 48h after treatment. Between the two cell lines, only 86 genes were found in common. Gene Ontology-based analysis revealed that the most significantly altered processes, regarding A549 cells, were DNA damage response and repair, mitotic relative functions, cell cycle and proliferation while in H157 cells, they were mainly related to transcription regulation, cholesterol and fatty acid biosynthesis, apoptosis, inflammatory response, cell cycle and adhesion. MNU induced distinct responses at the gene expression level in the above cell lines; in A549 cells, the contribution of pyroptosis in the MNU-induced cell death was further validated by caspase-1 western blotting and inhibition, DNA repair processes were down-regulated and a significant number of p53 downstream genes were up-regulated indicating the induction of a p53-, caspase-1-dependent cell death mechanism, while in H157 cells genes related to apoptosis were induced accompanied by down-regulation of pro-inflammatory genes. Our results support the use of S_N_1 methylating agents in platinum-based combination regimen against advanced NSCLC.
